# Efficacy and Safety of Transcutaneous Electrical Acupoint Stimulation (TEAS) for Postoperative Pain in Laparoscopy: A Systematic Review and Meta-Analysis of Randomized Controlled Trials

**DOI:** 10.1155/2022/9922879

**Published:** 2022-01-15

**Authors:** Dan Meng, Yifei Mao, Quan-mei Song, Chun-chun Yan, Qin-yu Zhao, Mengqi Yang, Guangxin Xiang, Yongmei Song

**Affiliations:** ^1^Institute of Literature and Culture of Traditional Chinese Medicine, Shandong University of Traditional Chinese Medicine, Jinan, Shandong 250355, China; ^2^Institute of Acupuncture, Moxibustion, and Massage, Shandong University of Traditional Chinese Medicine, Jinan, Shandong 250355, China; ^3^Institute of Traditional Chinese Medicine, Shandong University of Traditional Chinese Medicine, Jinan, Shandong 250355, China

## Abstract

**Objectives:**

This meta-analysis aimed to assess the efficacy and safety of transcutaneous acupoint electrical stimulation (TEAS) for postoperative pain in laparoscopy. The review has been registered on the “INPLASY” website and the registration number is INPLASY202150101.

**Methods:**

Relevant randomized controlled trials are selected from seven electronic databases (PubMed, the Cochrane Library, Embase, China National Knowledge Infrastructure, Chongqing VIP Information, WanFang Data, and Chinese Biomedical Database) from their inception up to November 30, 2020. Twenty-eight studies were included in this meta-analysis, and the statistical analyses and the exploration of heterogeneity sources were conducted by Stata 15.0 software. Besides, the bias assessment of the included studies was evaluated using the Cochrane risk of bias tool.

**Results:**

In total, 28 RCTs covering 2787 participants were included. The meta-analysis suggested that TEAS can effectively relieve pain in the short term after laparoscopy, reduce the postoperative consumption of rescue analgesics, improve the quality of life of patients, and shorten the length of hospitalization. And no serious adverse events are related to TEAS. Therefore, TEAS is relatively safe and efficacy for clinical application. The most used acupoints were Hegu (LI14), Neiguan (PC6), and Zusanli (ST36).

**Conclusions:**

TEAS can be recommended as a complementary and alternative therapy for the treatment of postoperative pain after laparoscopy. However, the included RCTs had some methodological limitations. Therefore, larger-size, more rigorous, and higher-quality RCTs are needed in the future to further explore the efficacy and safety of TEAS for postoperative pain after laparoscopy.

## 1. Introduction

With the promotion of the concept of minimally invasive surgery, the application of laparoscopy has gradually increased because of its advantages of small trauma to surrounding tissues, less intraoperative bleeding, rapid postoperative recovery, and a wide application range. However, various types of pain such as incision pain, visceral pain, and nonincision pain often occur after laparoscopy, among which shoulder and back pain, diaphragm pain, intercostal muscle pain, and other nonoperative incision pain are more common [1]. This is a serious complication after laparoscopy, which is called “postlaparoscopic pain syndrome” [2]. It may be related to the following factors: the inflammatory injury or traction of the phrenic nerve caused by the residual CO_2_ in the abdomen after the surgery, the proliferation of peritoneal inflammation induced by the acid environment in the abdomen, the humidity and temperature of the pneumoperitoneum, and the position and types of the surgery [3–7].

To some extent, postoperative pain may produce a negative effect on patients' physical activity, recovery time, quality of life, medical expenses, etc. Therefore, optimizing the management of postoperative acute pain is crucial for accelerating postoperative rehabilitation. Though a variety of analgesic methods, such as nonsteroidal anti-inflammatory drugs (NSAIDs) or COX-2 analgesic drugs, as well as epidural analgesia, patient-controlled intravenous analgesia (PCIA), and nerve block, have been widely used in the clinic, they can also produce more side effects. For example, opioids can lead to postoperative nausea and vomiting, skin pruritus, respiratory inhibition, and other adverse reactions [8]. Therefore, to reduce the side effects, many researchers began to turn to complementary and alternative therapies. One study [9] found that acupuncture and related techniques can significantly reduce postoperative pain score and opioid consumption and can be used as an effective auxiliary means of postoperative pain management. One study [10] reported that TEAS can significantly relieve postoperative pain and reduce opioid consumption on the first postoperative day. Another study [11] limited the mode of acupuncture to the way needling with penetration of the skin and only investigated the efficacy of such way on postoperative pain after laparoscopy and did not analyze the noninvasive acupoint stimulation mode.

Transcutaneous acupoint electrical stimulation (TEAS) is a noninvasive treatment formed by the gradual improvement of traditional acupuncture and moxibustion. During the operation, the specific low-frequency pulse current is input into the human body through the electrodes placed on the acupoints' surface and stimulates the acupoint to achieve a certain therapeutic effect, which combines the advantages of acupoint therapy and transcutaneous electric nerve stimulation (TENS). It can reduce the incidence of infection and bleeding, can be controlled or reused by patients and be operated by the operator after simple training, and has good treatment compliance. Many clinical randomized controlled trials (RCTs) have reported transdermal electrical stimulation is widely used in surgeries and plays a positive role in the treatment of postoperative pain [12–14]. In addition, pharmacological studies have shown that some medicinal plants are safe natural analgesics and have potential therapeutic advantages. For example, *Molineria capitulata* and *Spirulina platensis* have anti-inflammatory, antioxidant, and analgesic effects [15, 16]. *Bromelain* is a natural drug to alleviate the symptoms of arthritis (including joint pain and stiffness) [17]. It has an active therapeutic effect on immune system diseases. *Flavonoids* can play an auxiliary role in the treatment of pain in the digestive system and nervous system [18]. These studies may bring new clinical ideas to the treatment of postoperative pain. However, this time we are only going to evaluate the efficacy and safety of TEAS in the treatment of postoperative pain after laparoscopy, so we conducted this systematic review and meta-analysis. To our knowledge, this is the first meta-analysis focusing on the outcomes of TEAS in the treatment of postoperative pain after laparoscopy.

## 2. Materials and Methods

This systematic review and meta-analysis was performed in line with the Preferred Reporting Items for Systematic Reviews and Meta-Analyses (PRISMA) schema and was registered at INPLASY under registration number INPLASY202150101, and the corresponding protocol has been published in *Medicine* [19].

### 2.1. Data Sources and Search Strategy

A comprehensive search was carried out in PubMed, the Cochrane Library (CENTRAL), Embase, and four Chinese databases (China National Knowledge Infrastructure, Chongqing VIP Information, WanFang Data, and Chinese Biomedical Database) from their inception up to November 30, 2020. Because of the language restriction of our researchers, only studies published in English and Chinese were included. The search strategy consisted of Medical Subject Heading (Mesh) terms and free-text terms with logical operators. Asterisks are truncation symbols that can help us search for all designs with asterisks. We performed an initial search of PubMed as follows (Table 1): #1 “transcutaneous electrical acupoint stimulation” OR “transcutaneous acupoint electrical stimulation” OR “electr^*∗*^ stimulat^*∗*^” OR “electr^*∗*^ acustimul^*∗*^” OR “electroacupuncture^*∗*^” OR “electro-acupuncture” OR “TEAS”; #2 “Laparoscopy[Mesh]” OR “laparoscop^*∗*^” OR “coelioscop^*∗*^” OR “celioscop^*∗*^” OR “peritoneoscop^*∗*^”; #3 “Pain, Postoperative[Mesh]” OR “postoperative pain” OR “postoperative analgesi^*∗*^” OR “pain management” OR “ache^*∗*^” OR “suffering^*∗*^” OR “discomfort.” We also further searched the grey literature and the retrieved references to avoid omission. For the literature to be difficult to obtain the full text, we checked and identified the ongoing or unpublished studies through the World Health Organization International Clinical Trials Registry Platform (WHO ICTRP), Clinical Trials.gov, Chinese Clinical Trial Registry (Chi CTR), and the reference list of eligible RCTs. Two reviewers (Meng and Mao) independently screened the titles and abstracts for eligibility and examined the full text of the articles. Any discrepancies were resolved by consensus or after consulting a third party (Song).

### 2.2. Study Selection

#### 2.2.1. Inclusion Criteria of Studies

1. Participants: they were adults over 18 years old and underwent any kind of laparoscopy under general anesthesia. There are no restrictions on gender, race, occupation, location, malignancy of the disease, and many other aspects. When these patients underwent surgery other than laparoscopy, we only analyzed the pain indicators related to laparoscopy.
2. Type of study: randomized controlled trials (RCTs) in English or Chinese with no limitation of the blinding method.
3. Type of interventions: the intervention groups were treated with TEAS or TEAS combined with other therapy, such as patient-controlled intravenous analgesia (PCIA) or other anesthesia methods. The intervention time, frequency, waveform, acupoints, and course of treatment of TEAS are not limited. The control group were treated with sham-TEAS or blank control or combined with other treatment methods which are the same as the intervention group or combination of the above several methods.
4. Study outcomes: primary measures: (a) pain intensity: relevant overall postoperative pain using any scale, such as Visual Analogue Scale (VAS); (b) consumption of postoperative analgesics. Secondary measures: (a) quality of life after the surgery: assessed using validated scales, such as the pain score in QoL-40; (b) duration of hospitalization; (c) adverse events such as postoperative nausea, vomiting, pruritus, etc. Safety evaluation was assessed using the adverse event reported in the studies

#### 2.2.2. Exclusion Criteria of Studies

1. Non-RCTs, crossover trials, quasi-randomized trials, protocols, reviews, case reports, animal experimental research studies, ongoing trials, literature without full text, duplicate publications, and irrelative interventions and outcomes were excluded.
2. We excluded trials in which the controls underwent different frequency, waveform, intervention time, and other forms of TEAS compared with the intervention group.

### 2.3. Data Extraction and Quality Assessment

Data extraction was independently conducted by two investigators (Meng and Mao) according to the criteria above. Disagreements were resolved through discussion and consensus was reached with a third investigator (Yan). The two authors made a final judgment by reading the full text of the remaining articles. All available information related to our research was extracted from the included studies. The following data was extracted from the studies using a predesigned form: first author name, publication year, types of surgery, sample size, intervention, intervention measures and time, acupoints, waveform, electrical stimulation frequency, outcome measures, and adverse events. If the information was incomplete, we tried to acquire it by contacting the correspondent authors via e-mail or telephone.

The Cochrane risk of bias tool [20] was used to assess the methodological qualities of the included trials by the two reviewers (Meng and Mao). The contents included random sequence generation, allocation concealment, blinding of participants and personnel, blinding of outcome assessment, selective reporting, incomplete outcome data, and other risks of bias. The risk of bias was classified as “low-risk,” “high-risk,” or “unclear-risk.” Disagreements in this interpretation were resolved via consensus or after discussion with a third party (Song).

### 2.4. Data Analysis

Statistical analysis was performed with the Stata 15.0 software. *P* < 0.05 was considered statistically significant. For continuous variables, so the mean difference (MD) or standard mean difference (SWD) with 95% confidence interval (CI) was used for analysis. For dichotomous data, such as the rates of responders and adverse events, the relative risk (RR) with 95% confidence intervals (CIs) was utilized for analysis. In the multiarm RCTs, we only extracted the data related to our study. For studies that satisfied the predefined inclusion criteria with multiple intervention groups, which used different intervention duration or different acupoints, meta-analysis will be conducted carefully when merged the data into a unified intervention group data. The magnitude of heterogeneity was measured using heterogeneity index *I*^2^ statistic: when *I*^2^ < 50%, a fixed-effects model will be used for pooled data; when *I*^2^ ≥ 50%, a random-effects model was used. For each merged analysis, a heterogeneity test was performed using the chi-squared statistic. If *I*^2^ ≥ 50%, the synthesized studies were considered an indicator of a substantial level of heterogeneity. Subgroup or sensitivity analysis was performed to identify the cause. Subgroup analyses identified the possible factors that contributed to the heterogeneity, such as different intervention duration, interventions, types of surgery, and waveform. Funnel plots were introduced to detect the existence of potential publication bias within RCTs (*n* ≥ 10). If meta-analysis is not feasible, narrative descriptions are provided. If heterogeneity of significance found in the included studies cannot be explained by subgroup analysis, we will not conduct a meta-analysis.

## 3. Results

### 3.1. Search Results

**Figure 1:**
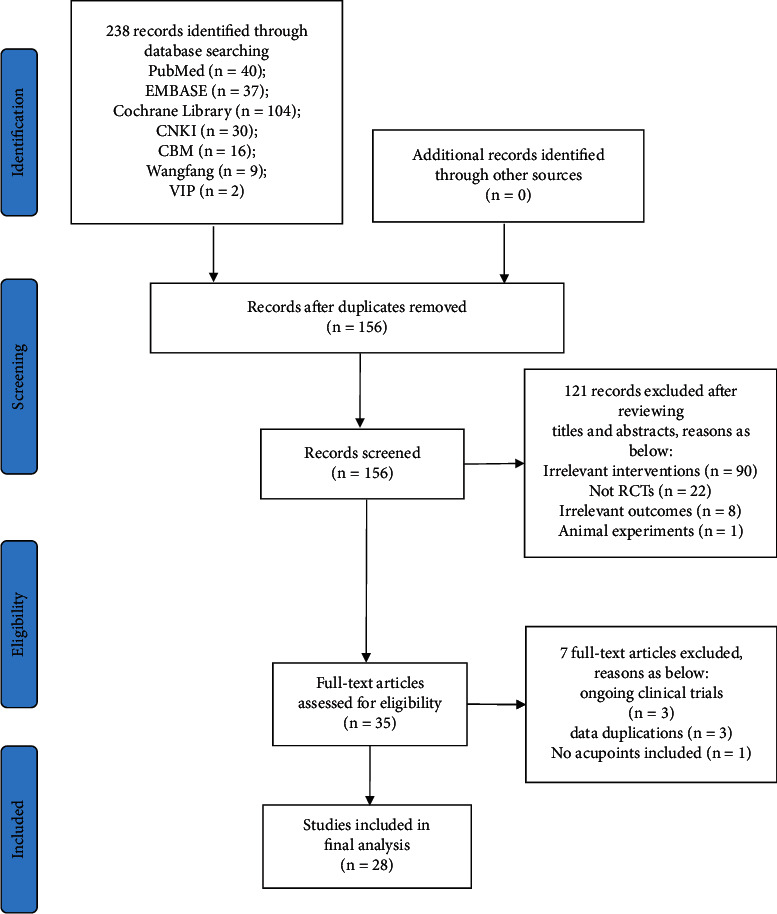
PRISMA flow diagram of the study process. PRISMA = preferred reporting items for systematic review and meta-analysis.

A total of 238 articles were identified during the initial study selection, 82 duplicates were removed, and 121 articles were excluded after reviewing abstracts and titles. Seven articles were excluded after reading the full text. For articles in which the data were repeatedly published, we choose the one with the higher quality and more related outcomes. Finally, 28 RCTs [21–48] met the eligibility criteria and were included in the systematic review. Standard general anesthesia was used in all trials. The PRISMA flow chart showed the study selection process in Figure 1.

### 3.2. Characteristics of Studies Included in the Review

In the included 28 trials [21–48], 4 [22, 23, 34, 47] were published in English, and the others were published in Chinese. There were 1627 participants in the intervention group and 1160 participants in the control group. All of the included studies were conducted in China and showed no significant difference within the baseline. The surgery types were different among these trials, much of them underwent gynecological surgery [22, 24, 26, 28, 33–35, 37, 40, 41, 43, 44, 46] and gastrointestinal surgery [26, 29–31, 42, 45, 47, 48]. In the treatment groups, 18 studies [21–38] adopted TEAS alone, 5 studies [39–43] adopted TEAS with PCIA/PCEA, and 5 studies [44–48] adopted TEAS combined with other treatments. In the control groups, 11 studies [21–31] adopted sham-TEAS, 7 studies [33–38, 44] adopted blank control, and 10 studies [32, 39–43, 45–48] adopted the same measures as intervention groups besides TEAS. There is a total of 14 acupoints, in which the most common acupoints are Zusanli (ST36), Nieguan (PC6), and Hegu (LI14). The intervention time was different and the duration from the time before anesthesia induction to the end of the operation was most commonly used. As we can see, the dilatational waveform and continuous waveform are commonly used. For stimulation frequency, 2/100 Hz and 2/10 Hz were popular among the trials. The main characteristics and the specific interventions of each identified study are presented in Table 2.

### 3.3. Quality Assessment

**Figure 2:**
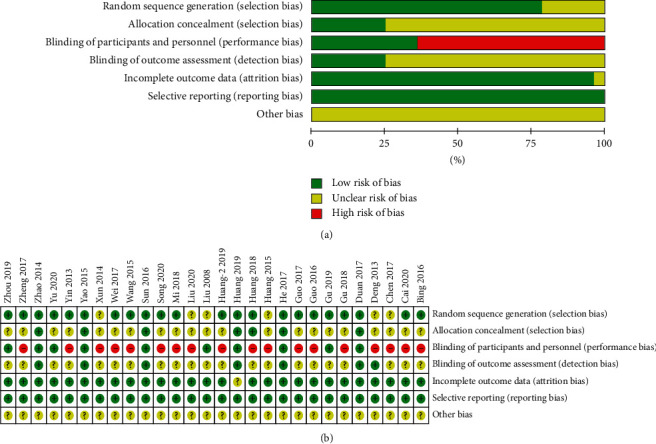
Risk of bias from included studies. (a) Risk of bias summary. Review authors' judgments about each risk of bias item for each included study. (b) Risk of bias graph. Review authors' judgments about each risk of bias item presented as percentages across all included studies.

Figure 2 summarizes the risk of bias in the 28 eligible RCTs, and the grade of the risk was recorded as “high-risk,” “low-risk,” and “unclear-risk.” In terms of random sequence generation, 23 studies [21–35, 39, 41–44, 46–48] were considered as “low-risk” for they reported explicit random sequence methods, such as random number table method or computer-generated random number while others [36–38, 40, 45] were considered as “unclear-risk” for they only mentioned “random” or “randomization” and did not report the specific random sequence method. In terms of the methods of allocation concealment, 7 studies [22, 24, 28, 32, 43, 47, 48] were considered as “low-risk” for they reported the method of assigning concealment, while others did not report it. 10 studies [22–24, 27–29, 32, 34, 43, 47] were considered as “low-risk” which performed a blinding method on subjects and operators. 7 studies [22, 24, 28, 32, 40, 43, 47] were considered as “low-risk” which performed a blinding method on results evaluators. 27 eligible RCTs reported the planned outcomes, so they were associated with a “low risk” of bias for blinding of selective reporting while one study [47] was considered as “unclear risk” for it did not report the predesigned outcomes in the research protocol. One study [47] did not report missing data and was considered as “unclear risk,” while other studies were considered as “low risk” for they reported the number and reasons of dropout members or no dropout members were in their studies. Additionally, the included 28 eligible RCTs did not report any details about potential sources of bias; therefore, they were considered as “unclear risk” in other biases.

### 3.4. Primary Outcomes

#### 3.4.1. Visual Analogue Scale (VAS)

Among the included studies, 8 studies were reported only in the form of graphs, without mean and standard deviation. However, our contact with the author is often unsuccessful. Therefore, the data are often unavailable and can only be excluded. One study [43] recorded the mean VAS score and the most painful VAS score within 24 h after surgery. One study [26] recorded the incidence of shoulder pain 48 h after surgery. A study [23] recorded the postoperative Verbal Rating Scale (VRS) at different times. One study [36] reported 2 cases of VAS ≥ 4 in postoperative adverse reactions. Due to the insufficient number of studies reporting these data, no meta-analysis could be completed. Seventeen studies [25, 27, 29–31, 34, 35, 37–42, 44–46, 48] recorded VAS scores at different times after the surgery. Therefore, to improve the validity of evidence, only studies (*n* ≥ 5 RCTs) reported VAS scores were analyzed to explore the analgesic effect of TEAS after laparoscopy.

**Figure 3:**
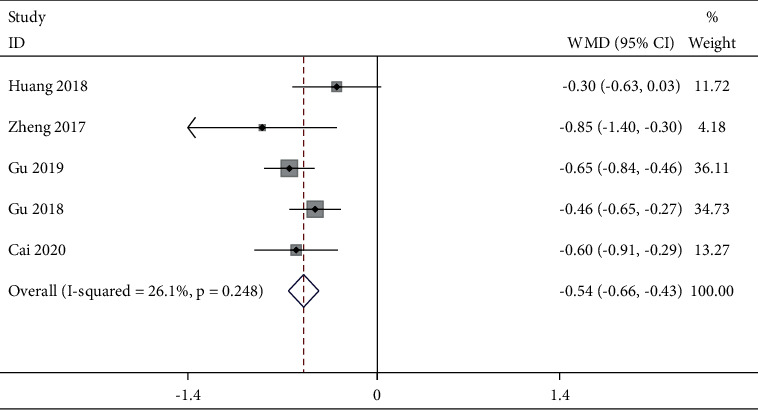
Forest plot of VAS for postoperative pain intensity at 4 h in TEAS compared with control.

**Figure 4:**
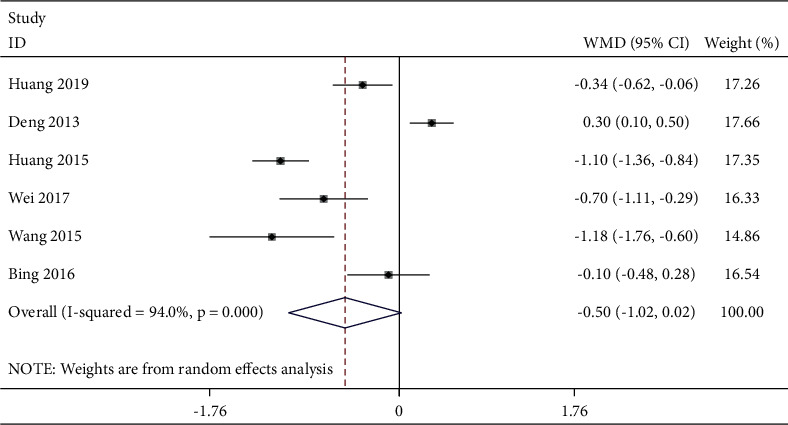
Forest plot of VAS for postoperative pain intensity at 6 h in TEAS compared with control.

**Figure 5:**
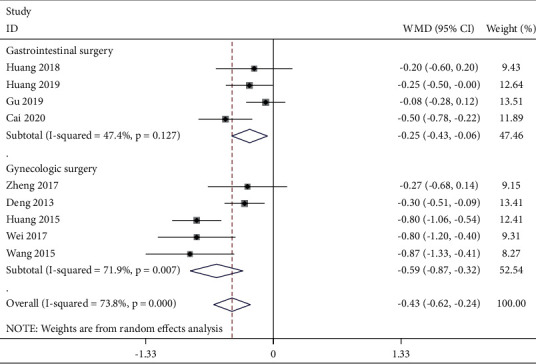
Forest plot of VAS for postoperative pain intensity at 12 h in TEAS compared with control.

**Figure 6:**
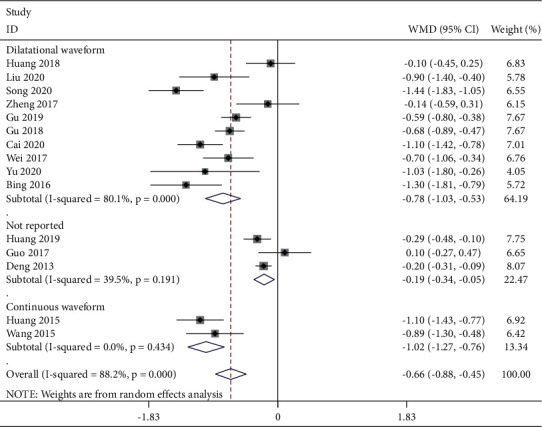
Forest plot of VAS for postoperative pain intensity at 24 h in TEAS compared with control.

Five studies reported a postoperative VAS score at 4 h (Figure 3). The fixed-effects model was used with good homogeneity (*P* = 0.248, *I*^2^ *=* 26.1%). The combined data showed that the TEAS group was effective in reducing pain at this time compared with the control group [WMD = −0.54, 95% CI (−0.66, −0.43),*P* < 0.001]. Six studies reported postoperative VAS scores at 6 h (Figure 4). Due to obvious heterogeneity (*P* < 0.001, *I*^2^ = 94.0%), a random-effects model was used and showed no statistical difference between the two groups. The combined data extracted from 9 studies related to postoperative VAS score 12 h showed obvious heterogeneity (*P* < 0.001, *I*^2^ = 73.8%), and a random-effects model was used. Based on the types of surgery, gastrointestinal surgery showed good intergroup homogeneity (*P* = 0.127, *I*^2^ = 47.4%). The subgroup analysis in favor of the TEAS group was effective in reducing pain at this time compared with the control group in the gastrointestinal surgery and gynecological surgery [WMD = −0.25, 95% CI (−0.43, −0.06), *P* = 0.009; WMD = −0.59, 95% CI (−0.87,−−0.32), *P* < 0.001] (Figure 5). Fifteen studies (Figure 6) provided data related to postoperative VAS score at 24 h and a random-effects model was used for a statistical analysis because of obvious heterogeneity (*P* < 0.001, *I*^2^ = 88.2%). Based on the waveform, we conducted a subgroup analysis. The homogeneity between continuous waveform groups is good (*P* = 0.434, *I*^2^ = 0%). The results showed significant pain reduction in favor of the TEAS group versus the control group at this time when applied continuous wave and dilatational wave [WMD = −1.02, 95% CI (−1.27, −0.76), *P* < 0.001; WMD = −0.78, 95% CI (−1.03, −0.53), *P* < 0.001].

#### 3.4.2. The Postoperative Consumption of Analgesics

**Figure 7:**
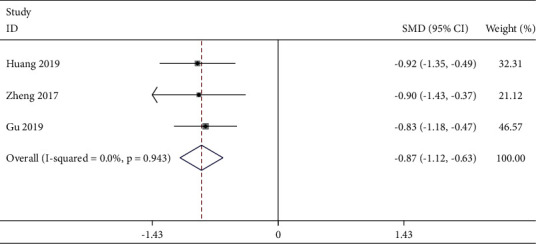
Forest plot of postoperative consumption of analgesics at 12 h in TEAS compared with control.

In our study, the consumption of postoperative analgesics after laparoscopy was used as another important indicator to evaluate the analgesic effect of TEAS. Six studies [21, 29, 30, 38, 41, 42] reported it. After our analysis, the postoperative consumption of analgesics 12 h was suitable for meta-analysis. Three studies [29, 30, 41] reported this outcome. The fixed-effects model (Figure 7) was used according to no significant homogeneity (*P* = 0.943, *I*^2^ = 0%). The combined data indicated that the postoperative consumption of analgesics was significantly lower in the TEAS group than that in the control group [SMD = −0.87, 95% CI (−1.12, −0.63), *P* < 0.001].

### 3.5. Secondary Outcomes

#### 3.5.1. QoR-40 (Quality of Recovery-40) Score

Five studies [21, 22, 34, 43] reported the quality of recovery after surgery, in which four studies used the QoR-40 scoring scale, and one study [28] used the QoR-15 scoring scale. Four studies [21, 22, 34, 43] reported postoperative QoR-40 scores at different times, in which one study [28] did not record in the form of mean ± standard deviation, so we excluded it. Based on the analysis of the data, the data of 24 h after surgery were considered suitable for meta-analysis.

**Figure 8:**
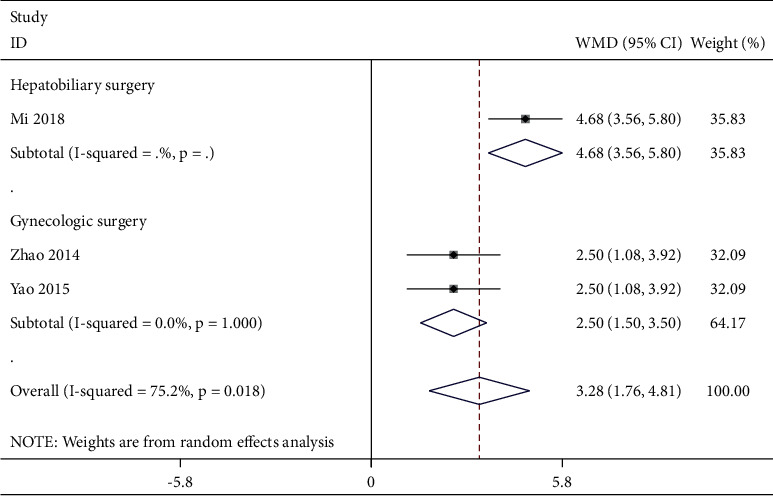
Forest plot of postoperative QoL-40 at 24 h in TEAS compared with control.

Three studies [21, 22, 43] reported this outcome. A random-effects model (Figure 8) was selected due to the obvious heterogeneity (*P* = 0.018, *I*^2^ *=* 75.2%). According to different types of surgery, the subgroup analysis was performed. It showed good homogeneity (*P* = 1.000, *I*^2^ = 0%) and significant improvement [WMD = 2.50, 95% CI (1.50, 3.50), *P* < 0.001] within the TEAS group who underwent gynecological surgery.

#### 3.5.2. Duration of Hospitalization

**Figure 9:**
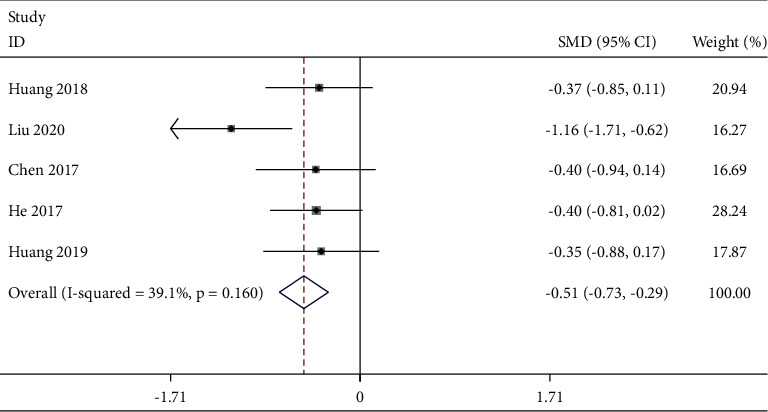
Forest plot of duration of hospitalization in TEAS compared with control.

Five studies [24, 38, 45, 47, 48] reported the duration of hospitalization. The fixed-effects model (Figure 9) was used with good homogeneity (*P* = 0.16, *I*^2^ = 39.1%). Meta-analysis results indicated that the duration of hospitalization in the TEAS group was shorter than that in the control group [SMD = −0.51, 95% CI (−0.73, −0.29), *P* < 0.001].

#### 3.5.3. Adverse Events

Adverse events are the main evidence of safety evaluation. The adverse events in our study refer to a series of complications after the operation (e.g., nausea, vomiting, dizziness, pruritus, respiratory depression, etc.). In the 28 included studies, 6 [21, 28, 32, 36, 38, 44] of which reported postoperative adverse events, while the remaining studies did not mention anything about safety evaluation. Mi et al. [21] recorded the incidence of nausea and vomiting in the treatment and control groups at different time points after surgery. And the comparison between groups at 4 h and 8 h after surgery showed statistical advantage (*P* < 0.05), but there was no significant difference at 24 h and 48 h after surgery. The results of Chen et al. [38] showed, that compared with the control group, there was no case of vomiting and shivering in the treatment group, and there were only two cases of nausea. The results of 4 studies [28, 32, 36, 44] showed that no adverse events were observed throughout the procession of TEAS.

### 3.6. Sensitivity Analyses and Publication Bias

**Figure 10:**
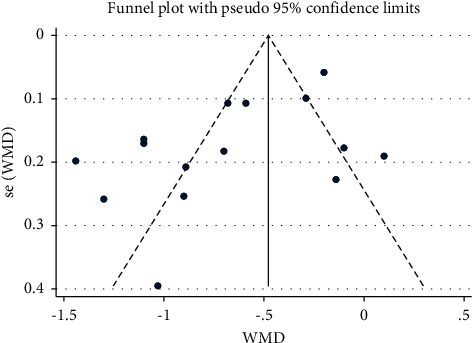
Funnel plot of publication bias.

**Figure 11:**
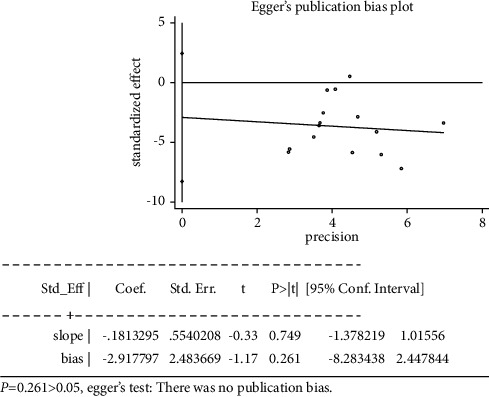
Egger's test of publication bias.

To assess the stability of the study results, our study performed a sensitivity analysis on the meta-analysis one by one. It showed that the results of each meta-analysis were stable. A funnel plot was used to assess publication bias based on the VAS score at 24 hours after the surgery *n* ≥ 10 RCTs) (Figure 10). It showed that most of the points were asymmetrically distributed around, indicating that there maybe have been some publication bias. Consistently, Egger's test (Figure 11) suggested there is no publication bias in the RCTs (*P* = 0.261 > 0.05).

## 4. Discussion

Enhanced recovery after surgery (ERAS) advocates optimizing multiple approaches to perioperative pain management [49]. Postoperative analgesia is an important part of ERAS [50]. Nonincision pain is the most common complaint after laparoscopy, with an incidence of about 70%∼80% [51]. The continuous administration of analgesics will reduce the pharmacological effectiveness [52], and a series of side effects will be produced by opioids. Therefore, there is an urgent need for optimized strategies to relieve postoperative pain after laparoscopy and improve the postoperative quality of life of patients.

To systematically assess the efficacy and safety of TEAS for postoperative pain after laparoscopy, our study conducted a systematic review and meta-analysis of 28 RCTs involving 2787 patients. Our meta-analysis suggests that TEAS can effectively relieve pain in the short term after laparoscopy, reduce the postoperative consumption of rescue analgesics, improve the quality of life of patients, and shorten the length of hospitalization. Therefore, TEAS can be recommended as a complementary and alternative therapy for the treatment of postoperative pain after laparoscopy. Besides, no serious adverse events related to TEAS and the occurrence of treatment-related safety problems were reported in the included studies. Therefore, we could think cautiously that TEAS is relatively safe and efficacy for clinical application.

A previous study suggested that epidural anesthesia can reduce postoperative pain scores in abdominal surgery [53], so it is often regarded as a confounding factor for electroacupuncture or electrical stimulation to exert postoperative analgesia [54]. Therefore, our study only chose the operation of laparoscopy under general anesthesia, to avoid the interference of more potential factors. Pain intensity was selected as the outcome for it plays an important role in pain assessment and management [55]. VAS is commonly used in pain evaluation. Previous studies [56] have suggested that the subjectivity and multidimensionality of pain experience produce individual differences in pain assessment. Some studies showed that analgesic requirements are controlled by healthcare providers and are directly affected by the types of surgery and the economic status of patients. Analgesic consumption is not a particularly reliable indicator of the effectiveness of acupoint stimulation [57]. Therefore, more objective indicators, such as the metabolism of pain substances in the blood, will be needed for postoperative pain assessment in the future.

Besides, we did not categorize different types of pain, which may affect the results. Therefore, a more rigorous experimental design is needed to confirm the results of our study. We found that TEAS was effective in alleviating pain intensity at 4h, 12h, and 24h after laparoscopy, although this may depend on the type of surgery and waveform of the electrical stimulation device. TEAS can also effectively reduce the consumption of analgesics 12 h after surgery, but the sample size included in this study was small, and the pharmacological mechanism of different analgesics was not fully considered. This indicates that TEAS can effectively relieve postoperative pain in the short term. However, in the process of future studies, more objective large-scale RCTs are still needed to provide more evidence support, and more attention should be paid to the standardized research program of TEAS and the follow-up of long-term efficacy.

At present, the analgesic mechanism of TEAS is not clear. Some studies [58–60] believe that the treatment process can cause the release of neurotransmitters in some regions of the spinal cord and brain, which increases the endogenous analgesic substances such as opioid peptides, strong kephalins, enkephalins, and acetylcholine, thus raising the pain threshold. It has been suggested that electrical stimulation can block pain signals by activating the descending inhibitory system in the brain stem [61]. It has been suggested that electrical stimulation activates different functional areas of the cerebral cortex, thereby reducing the body's sensitivity to painful stimuli by affecting the corresponding innervation of the skin [62].

For patients who have experienced laparoscopy, the body function is in a relatively weak state, resulting in patients with insufficient strength, blood stasis, and reduced meridians, resulting in postoperative pain symptoms. According to the traditional meridian theory of traditional Chinese medicine, the regulating effect of acupuncture and moxibustion is reflected not only in regulating the movement of Qi and blood, making the meridians and collaterals smooth, but also in regulating the ups and downs of Qi and blood, so that the organs and collaterals are supported by Qi and blood, and correcting the partial decline of the body. Acupoints are special parts of the viscera by draining Qi into the body surface. Electrical stimulation on the acupoints can play the role of regulating Qi and regulating blood, draining meridians, and dredging collaterals. Hegu (LI14), Neiguan (PC6), and Zusanli (ST36) are important acupoints for analgesia in meridian theory. Both ancient literature and modern studies show that Hegu (LI14) is good at relieving pain [63]. The experimental study [61] showed that the plasma concentration of 5-hydroxytryptamine (5-HT) decreased after electroacupuncture of Hegu (LI14) in rabbits, to achieve the purpose of analgesia. It can play an analgesic role in many ways, such as increasing the body pain threshold, alleviating microvascular spasms, and influencing the frontal cortex to participate in the discharge of the pain unit in the ventrolateral thalamic nucleus [64]. Besides, it has been reported to be effective in treating acute pain in various parts of the body [65]. Neiguan (PC6) has the effect of acute pain in various parts of the body [66]. Zusanli (ST36) has the functions of health care, immune regulation, strengthening and dispelling pathogenic factors, and regulating the spleen and stomach. Electroacupuncture stimulation of bilateral Zusanli (ST36) in rats can stimulate the parasympathetic nerve and cholinergic pathway, which can effectively promote the recovery of gastrointestinal function in rats [67, 68]. These acupoints are convenient and accurate, are easy to implement, and have good treatment compliance.

The QoR-40 scale is designed to evaluate the quality of postoperative recovery. Its effectiveness has been verified clinically, and it is widely used to evaluate the quality of postoperative recovery [69]. The duration of hospitalization is also an important indicator of postoperative recovery. Our results are similar to those of the previous studies [54], which reported that TEA was effective in reducing the duration of hospitalization (MD = 1.30, 95% CI (−2.10, −0.51), *I*^2^ = 32%, REM). Although this study has found that TEAS can improve postoperative quality of life and shorten the duration of hospitalization, more large RCTs with objective results are needed to confirm these findings.

For the aspects of the waveform and electrical stimulation frequency, the included studies mostly adopted dilatational waveform with 2/100 Hz. In terms of analgesia, a previous study [70] has suggested that the analgesic effect of alternating stimulation of density wave is better than that of simultaneous stimulation of different frequencies. TEAS used in 2/100 Hz induced the release of all opioid peptides and peptides with synergistic effects [71]. It can be seen that these research results are consistent with our data results. For the intervention time, most of the included studies were conducted from the time before anesthesia induction to after surgery. One study [72] has found that preoperative electroacupuncture therapy can relieve the tension and fear of patients and, to a certain extent, reduce the number of postoperative opioids for patients with mental stress. It has been shown that preoperative acupoint therapy interventions can reduce the need for opioids and opioid-related side effects after surgery [73, 74]. However, one study [75] found that intraoperative treatment did not improve postoperative pain and did not reduce the amount of opioids needed. Besides, the brain will produce different responses with the different timing of acupuncture treatment, thus affecting the therapeutic effect [76]. Therefore, it is suggested that the study on the timing of TEAS intervention should be strengthened and the treatment plan should be improved.

## 5. Limitations

It must be acknowledged that our meta-analysis has several limitations. (1) The intervention time, waveform, and frequency of electrical stimulation, acupoints, and other aspects of TEAS treatment are not uniform due to the different clinical experiences. At the same time, the treatment regimens in the control group were not uniform, which may affect the accuracy of the effectiveness assessment. (2) Our review may be affected by a high degree of heterogeneity, and it is difficult to find out the main reason that affects the efficacy of TEAS. (3) We did not distinguish between different types of pain and did not summarize the optimal TEAS treatment for postoperative laparoscopic pain. (4) Most RCTs were conducted in China, which may lead to publication bias and affect the validity and reliability of the system evaluation. (5) The methodological deficiencies and small sample size of the included trials may overestimate the efficacy to some extent. (6) No long-term follow-up information has been reported, so long-term efficacy remains to be confirmed.

## 6. Conclusions

In this systematic review and meta-analysis, TEAS can be cautiously considered as a treatment for postoperative pain after laparoscopy, which is effective in alleviating pain in the short term, reducing the use of remedial analgesics, and improving postoperative quality of life and length of hospital stay. TEAS is a noninvasive and easy-to-operate treatment, which can be operated after simple training, low application price, and good treatment compliance. It is worthy of clinical promotion and application. Although this study has limitations, it provides a direction and basis for future research. Due to the methodological deficiencies of the included RCTs, larger-size, more rigorous, and higher-quality RCTs are needed in the future to further explore the efficacy of TEAS in the treatment of postoperative pain after laparoscopy. Therefore, it is necessary to further optimize the treatment plan in terms of intervention time, electrical stimulation frequency and waveform, and acupoint selection. The long-term therapeutic effect of TEAS needs further studies determined by the observation of prolonged follow-up time. Besides, more attention should be paid to the economic impact of TEAS in the health care system.

## Figures and Tables

**Table 1:** Search strategy in PubMed up till November 30, 2020 (similar search run in other databases).

#1	“transcutaneous electrical acupoint stimulation” OR “transcutaneous acupoint electrical stimulation” OR “electr∗ stimulat∗” OR “electr∗ acustimul∗” OR “electroacupuncture∗” OR “electro-acupuncture” OR “TEAS”
#2	“Laparoscopy[Mesh]” OR “laparoscop∗” OR “coelioscop∗” OR “celioscop∗” OR “peritoneoscop∗”
#3	“Pain, Postoperative[Mesh]” OR “postoperative pain” OR “postoperative analgesi∗” OR “pain management” OR “ache∗” OR “suffering∗” OR “discomfort”
#4	#1 AND #2 AND #3

**Table 2:** Characteristics of the included studies.

No.	Study ID	Types of surgery	Intervention time	Sample size(T/C)	Intervention measures		Outcomes	Acupoints	Waveforms	Electrical frequency
					Intervention Group	Control Group				
1	Mi et al. 2018 [21]	Hepatobiliary surgery	Before induction of anesthesia—the end of surgery	50/50	TEAS	Sham-TEAS	②③	LI14,PC6,ST36	Dilatational waveform	2/100 Hz
2	Yao et al. 2015 [22]	Gynecologic surgery	Before induction of anesthesia—the beginning of surgery	35/36	TEAS	Sham-TEAS	①②③	LI14,PC6,ST36,SP6	Dilatational waveform	2/10 Hz
3	Liu et al. 2008 [23]	Hepatobiliary surgery	Before induction of anesthesia—the end of surgery	48/48	TEAS	Sham-TEAS	④	PC6	Dilatational waveform	2/100 Hz
4	He 2017 [24]	Gynecologic surgery	S-TEAS: before anesthesia induction—the beginning of anesthesia; L-TEAS: before induction of anesthesia—the end of surgery	34, 33/34	TEAS	Sham-TEAS	①	LI14, ST36	Dilatational waveform	2/10 Hz
5	Cai et al. 2020 [25]	Gastrointestinal surgery	Before induction of anesthesia—the beginning of surgery	24/25	TEAS	Sham-TEAS	①	ST36,PC6	Dilatational waveform	2/15 Hz
6	Guo et al. 2016 [26]	Gynecologic surgery	Before induction of anesthesia—the end of surgery	30/30	TEAS	Sham-TEAS	⑤	PC6,ST36	Dilatational waveform	2/100 Hz
7	Zhou 2019 [27]	General surgery& Gynecologic surgery	Before induction of anesthesia—the end of surgery	32/32	TEAS	Sham-TEAS	①	SP6,LI14	Dilatational waveform	2/100 Hz
8	Duan 2017 [28]	Gynecologic surgery	Before induction of anesthesia—the end of surgery	64, 65, 63/64	Group-D:TEAS (double acupoints); Group-P, S (single acupoint)	Sham-TEAS	①③	groupD:PC6,ST36, group-P:PC6, group-S:ST36	Dilatational waveform	2/10 Hz
9	Gu 2019 [29]	Gastrointestinal surgery	S-TEAS, L-TEAS: the beginning of the operation—the end of the operation	49, 49/48	S-TEAS+PCIA；L-TEAS+PCIA	Sham-TEAS	①②	ST36,PC6	Dilatational waveform	2/100 Hz
10	Huang and Liu 2019 [30]	Gastrointestinal surgery	30 min before induction of anesthesia and lasting for 40 min	46/46	TEAS	Sham-TEAS	②	GV20,PC6,ST36,SP6	not reported	not reported
11	Song 2020 [31]	Gastrointestinal surgery	Before induction of anesthesia—the end of surgery	25/25	TEAS	Sham-TEAS	①	LI14,PC6	Dilatational waveform	5/100 Hz
12	Sun et al. 2016 [32]	Hepatobiliary surgery& Gynecologic surgery	Each perioperative treatment lasted 30 min	91, 91, 89/90	Group-A, B, C (different intervention time)	Sham-TEAS	①②	LI14,PC6	Dilatational waveform	2/100 Hz
13	Yin et al. 2013 [33]	Gynecologic surgery	Before induction of anesthesia—the end of surgery	30/30	TEAS	Blank control	①	ST36,ST34	Continuous waveform	2Hz
14	Yu et al. 2020 [34]	Gynecologic surgery	Before induction of anesthesia—the beginning of surgery	30/30	TEAS	Blank control	①②③	GV20,GV29,ST36,PC6	Dilatational waveform	2/100 Hz
15	Wang and Zhang 2015 [35]	Gynecologic surgery	Before induction of anesthesia—the end of surgery	58/58	TEAS	Blank control	①	ST34,ST36	Continuous waveform	2Hz
16	Xun et al. 2014 [36]	Hepatobiliary surgery	Before induction of anesthesia—the end of surgery	25/25	TEAS	Blank control	⑤	PC6,ST36	Dilatational waveform	2/100 Hz
17	Huang 2015 [37]	Gynecologic surgery	Before induction of anesthesia—the end of surgery	50/50	TEAS	Blank control	①	ST36,ST34	Continuous waveform	2Hz
18	Chen et al. 2017 [38]	General surgery	Before anesthesia induction—the beginning of anesthesia	20, 20/20	Group A: TEAS; group B: TEAS (different acupoints)	Blank control	①②	group-A:PC6,ST36; group- B:PC6,ST36,BL32,BL34	Dilatational waveform	2/100 Hz
19	Guo et al. 2017 [39]	Hepatobiliary surgery	Before anesthesia induction—after surgery	40/40	TEAS + PCIA	PCIA	①	EX-HN1,HT7,PC6	not reported	2/100 Hz
20	Deng et al. 2013 [40]	Gynecologic surgery	Before induction of anesthesia—the end of surgery	100/100	TEAS + PCEA	PCEA	①	PC6,ST36	not reported	2/100 Hz
21	Zheng et al. 2017 [41]	Gynecologic surgery	after the surgery	30/30	TEAS + PCIA	PCIA	①②⑤	LI14,PC6,ST36,SP6	Dilatational waveform	2/100 Hz
22	Gu et al. 2018 [42]	Gastrointestinal surgery	S-TEAS: the induction of anesthesia—the end of surgery; L-TEAS:before anesthesia induction—after surgery	60, 60/60	S-TEAS+PCIA;L-TEAS+PCIA	Sham-TEAS + PCIA	①②	ST36,PC6	Dilatational waveform	2/100 Hz
23	Zhao 2014 [43]	Gynecologic surgery	Before induction of anesthesia—the beginning of surgery	35/36	TEAS + PCIA	Sham-TEAS + PCIA	①②③	LI14,PC6,ST36,SP6	Dilatational waveform	2/10 Hz
24	Wei et al. 2017 [44]	Gynecologic surgery	Before induction of anesthesia—the end of surgery	30/30	TEAS + analgesics	analgesics	①	ST36,ST34	Dilatational waveform	2/100 Hz
25	Liu et al. 2020 [45]	Gastrointestinal surgery	Before anesthesia induction—after surgery	30/30	TEAS + standard analgesic regimen	standard analgesic regimen	①	LI14,PC8,ST36	Dilatational waveform	2/100 Hz
26	Bing et al. 2016 [46]	Gynecologic surgery	after the surgery	30/30	TEAS + postoperative routine nursing	postoperative routine nursing	①	GB21,BL12,GV6, GV4, etc.	Dilatational waveform	0.02 Hz
27	Huang et al. 2019 [47]	Gastrointestinal surgery	Before induction of anesthesia—the end of surgery	29/28	TEAS + TAP + PCIA	TAP + PCIA	①	ST36	Dilatational waveform	2/10 Hz
28	Huang 2018 [48]	Gastrointestinal surgery	Before induction of anesthesia—the end of surgery	32/35	TEAS + TAP + PCIA	TAP + PCIA	①	ST36	Dilatational waveform	2/10 Hz

Explanation: Outcomes: ① VAS values at different time points after operation; ② consumption of analgesics; ③ QoR-40 scare score; ④ VRS values at different time points after operation; ⑤ incidence of postoperative pain; acupoints: Zusanli ST36, Neiguan PC6, Hegu LI14, Sanyinjiao SP6, Baihui GV20, Liangqiu ST34, Yintang GV29, Ciliao BL32, Xialiao BL34, Shenmen HT7, Laogong PC8, Guanyuan GV4, Qihai GV6, Jianjing GB21, Fengmen BL12, Sishencong EX-HN1.

## Data Availability

(1) This systematic review and meta-analysis was performed in line with the Preferred Reporting Items for Systematic Reviews and Meta-Analyses (PRISMA) schema and was registered at INPLASY under registration number INPLASY202150101; the hyperlink is https://inplasy.com/inplasy-2021-5-0101/. (2) The corresponding protocol has been published in Medicine; the doi is 10.1097/MD.0000000000026348.

## Supplementary Materials

Supplementary Materials In the supplementary files, there are seven sensitivity analyses, which are sensitivity analysis of VAS for postoperative 4 h, 6 h, 12 h, and 24 h, sensitivity analysis of QoR-40, sensitivity analysis of postoperative consumption of analgesics, and sensitivity analysis of duration of hospitalization. Sensitivity analysis can also be understood as robustness analysis, which is an important method mainly used to evaluate the robustness and reliability of the combined results of the meta-analysis. It is a common sensitivity analysis method to remove each included study one by one and then merge the effect quantity, changing the inclusion and exclusion criteria or removing a certain type of literature and then merging the effect quantity. Through the sensitivity analysis of this study, we can confirm that all results are stable and reliable.
