# Supplementary material for: Efficacy and Safety of Transcutaneous Electrical Acupoint Stimulation (TEAS) for Postoperative Pain in Laparoscopy: A Systematic Review and Meta-Analysis of Randomized Controlled Trials
Source: Evid Based Complement Alternat Med. 2022 Jan 15;2022:9922879. doi: 10.1155/2022/9922879 (PMC8783713; doi:10.1155/2022/9922879)
Supplement: Supplementary Materials — In the supplementary files, there are seven sensitivity analyses, which are sensitivity analysis of VAS for postoperative 4 h, 6 h, 12 h, and 24 h, sensitivity analysis of QoR-40, sensitivity analysis of postoperative consumption of analgesics, and sensitivity analysis of duration of hospitalization. Sensitivity analysis can also be understood as robustness analysis, which is an important method mainly used to evaluate the robustness and reliability of the combined results of the meta-analysis. It is a common sensitivity analysis method to remove each included study one by one and then merge the effect quantity, changing the inclusion and exclusion criteria or removing a certain type of literature and then merging the effect quantity. Through the sensitivity analysis of this study, we can confirm that all results are stable and reliable. [file 9922879.f1.zip › 9922879.f1/INPLASY202150101.pdf]

INPLASY - International Platform of Registered Systematic Review and Meta-analysis Protocols

INVOICE PUBLICATION FEES - $20.00

Billing Information

Dan Meng
Shandong University of Traditional Chinese Medicine, China
mengdan1993@sina.com

PAID

Payment Details

Payment Method: WeChat Pay
Status: Authorized

Invoice Number: **202150101**

Protocol: The Efficacy and Safety of Transcutaneous Electrical Acupoint Stimulation (TEAS) for Postoperative Pain in Laparoscopy: A Protocol for Systematic Review and Meta-Analysis of Randomized Controlled Trials

Date: 28 May 2021
E-mail: editorialmanager@inplasy.com
Address - INPLASY, Inc. 651 N Broad St, Suite 206, Middletown, DE 19709 US.
