# Supplementary material for: Efficacy and Safety of Transcutaneous Electrical Acupoint Stimulation (TEAS) for Postoperative Pain in Laparoscopy: A Systematic Review and Meta-Analysis of Randomized Controlled Trials
Source: Evid Based Complement Alternat Med. 2022 Jan 15;2022:9922879. doi: 10.1155/2022/9922879 (PMC8783713; doi:10.1155/2022/9922879)
Supplement: Supplementary Materials — In the supplementary files, there are seven sensitivity analyses, which are sensitivity analysis of VAS for postoperative 4 h, 6 h, 12 h, and 24 h, sensitivity analysis of QoR-40, sensitivity analysis of postoperative consumption of analgesics, and sensitivity analysis of duration of hospitalization. Sensitivity analysis can also be understood as robustness analysis, which is an important method mainly used to evaluate the robustness and reliability of the combined results of the meta-analysis. It is a common sensitivity analysis method to remove each included study one by one and then merge the effect quantity, changing the inclusion and exclusion criteria or removing a certain type of literature and then merging the effect quantity. Through the sensitivity analysis of this study, we can confirm that all results are stable and reliable. [file 9922879.f1.zip › 9922879.f1/Medicine protocol.pdf]

Study Protocol Systematic Review

Medicine®

OPEN

The efficacy and safety of transcutaneous electrical acupoint stimulation (TEAS) for postoperative pain in laparoscopy

A protocol for systematic review and meta-analysis of randomized controlled trials

Dan Meng, MD[a], Yifei Mao, MD[a], Quanmei Song, MM[b], Chunchun Yan, MD[b], Qinyu Zhao, MD[b], Mengqi Yang, MD[c], Yongmei Song, MD[a,*]

Abstract

Background: With the promotion of the concept of "minimally invasive" surgery, the advantages of laparoscopic surgery are increasingly manifested. However, the postoperative pain of laparoscopic surgery brings difficulties and challenges to patients' rehabilitation. Transcutaneous electrical acupoint stimulation (TEAS) is a non-invasive treatment, which can exert the dual efficacy of acupuncture and electrical stimulation. The efficacy and safety of TEAS for postoperative pain after laparoscopy based on randomized controlled trials (RCTs) need to further evaluate.

Methods: A comprehensive and systematic literature searching will mainly perform on 7 electronic databases (PubMed, the Cochrane Library, Embase, China National Knowledge Infrastructure, Chongqing VIP Information, WanFang Data, and Chinese Biomedical Database) from their inception up to November 30, 2020. We will also search for ongoing or unpublished studies from other websites (eg, PROSPERO, ClinicalTrials.gov, and Chinese Clinical Trial Registry) and do manual retrieval for potential gray literature. Only the relevant RCTs published in English or Chinese were included. Two independent investigators will independently complete literature selection, assessment of risk bias, and data extraction, the disagreements will be discussed with the third party for final decisions. The primary outcome measures: the pain intensity (eg, VAS) and the consumption of postoperative analgesics. The secondary outcome measures: the postoperative quality of life, the duration of hospitalization, and the incidence of adverse reactions and serious events. Assessment of bias risk will follow the Cochrane risk of bias tool. Data processing will be conducted by Stata 15.0 software.

Results: We will evaluate the efficacy and safety of TEAS for postoperative pain after laparoscopy based on RCTs.

Conclusion: This study can provide more comprehensive and strong evidence of whether TEAS is efficacy and safe for postoperative pain in laparoscopic surgery.

Abbreviations: CI = confidence interval, MD = mean difference, PRISMA-P = Preferred Reporting Items for Systematic Reviews and Meta-Analysis Protocol, RCTs = randomized controlled trials, TEAS = transcutaneous electrical acupoint stimulation, VAS = visual analogue scale.

Keywords: postoperative pain, protocol, systematic review and meta-analysis, transcutaneous electrical acupoint Stimulation

The research has been registered and approved on the INPLASY website. The registration number is INPLASY202150101.

Ethics approval is not required in our systematic review and meta-analysis which is based on published randomized clinical trials.

Our research object is literature, so no ethical review approval is required.

This research is funded by the Fourth Batch of National Excellent Clinical Talents of Traditional Chinese Medicine Research and Training Program (National Education of Traditional Chinese Medicine [2017] No.24) and Key Laboratory of Classical Theory of Traditional Chinese Medicine, Ministry of Education, Shandong University of Traditional Chinese Medicine, Jinan, 250355, China

The authors have no conflicts of interest to disclose.

The datasets generated during and/or analyzed during the present study are not publicly available but are available from the corresponding author on reasonable request.

[a] Institute of Literature and Culture of Traditional Chinese Medicine, [b] Institute of Acupuncture, Moxibustion, and Massage, [c] Institute of traditional Chinese medicine, Shandong University of Traditional Chinese Medicine, Jinan, Shandong 250355, China.

[*] Correspondence: Yongmei Song, Institute of Literature and Culture of Traditional Chinese Medicine, Shandong University of Traditional Chinese Medicine, 4655 University Road, University Science Park, Changqing District, Jinan, Shandong Province, China (e-mail: Songym0200@163.com).

Copyright © 2021 the Author(s). Published by Wolters Kluwer Health, Inc.
This is an open access article distributed under the Creative Commons Attribution License 4.0 (CCBY), which permits unrestricted use, distribution, and reproduction in any medium, provided the original work is properly cited.

How to cite this article: Meng D, Mao Y, Song Q, Yan C, Zhao Q, Yang M, Song Y. The efficacy and safety of transcutaneous electrical acupoint stimulation (TEAS) for postoperative pain in laparoscopy: A protocol for systematic review and meta-analysis of randomized controlled trials. Medicine 2021;100:25(e26348).

Received: 31 May 2021 / Accepted: 1 June 2021

http://dx.doi.org/10.1097/MD.0000000000026348

1. Introduction

Enhanced recovery after surgery recommended that the optimized multiple analgesic methods could be applied to perioperative pain management.[1,2] Although laparoscopic surgery has certain advantages in minimally invasive surgery, postoperative incision pain, visceral pain, and non-incision pain have brought great difficulties to the postoperative rehabilitation of patients. Although a variety of analgesic approaches have been widely used in clinical practice, they are followed by more side effects and adverse events, such as the use of opioids that can lead to the occurrence of nausea and vomiting, skin itching, and respiratory depression.[3] Therefore, to optimize the management of postoperative pain, postoperative rehabilitation began to shift to complementary and alternative medicine.

Transcutaneous electrical acupoint stimulation (TEAS) is a non-invasive treatment with high compliance. It acts on the corresponding acupoints through low-frequency pulsed electrical stimulation, thus exerting the dual efficacy of acupoint acupuncture and transcutaneous electrical nerve stimulation. A previous study[4] have shown that TEAS can improve the intensity of postoperative pain and significantly reduce the use of opioid on the first day after the surgery. Another study only intended to explore the efficacy of acupuncture (needling with penetration of the skin) on postoperative pain of laparoscopy but did not intend to evaluate the effect of non-invasive treatment. In recent years, TEAS has been widely used in cholecystectomy, colorectal cancer resection, ovarian cyst resection, and other surgical processes, and has a positive effect on the treatment of postoperative pain.[5–7] However, there has been no study to perform a meta-analysis on the efficacy of TEAS for postoperative pain after laparoscopy. Our study plan to evaluate the efficacy and safety of TEAS for the treatment of postoperative pain after laparoscopy based on randomized controlled trials (RCTs), to provide more evidence to support the management of postoperative pain.

2. Methods

2.1. Study registration

This systematic review protocol has been registered on the website of INPLASY. The approved registration number is INPLASY202150101.

2.2. Inclusion criteria for this study

2.2.1. Types of studies. All RCTs with TEAS for laparoscopic postoperative pain were included. Considering the language limitations of the study investigator, only relevant RCTs published in English or Chinese will be included. The non-randomized clinical trials, case reports, reviews, protocols, animal experimental researches, and ongoing or uncompleted trials will be excluded.

2.2.2. Types of participants. Patients who underwent laparoscopic surgery under general anesthesia with no limitation of the types of surgery will be included in our study. There are no restrictions on age, gender, race, education, socioeconomic status, types of disease, etc.

2.2.3. Type of interventions. The treatment group underwent TEAS or TEAS combined with multiple antalgic therapies, such as patient-controlled intravenous analgesia, postoperative continued epidural analgesia, or other analgesics. There are no restrictions on intervention time, frequency, waveform, acupoint, course of treatment, etc. The control group treated with sham-TEAS, blank control, or the same intervention as the treatment group other than TEAS will also be included. However, when the control group underwent different frequency, waveform, intervention time, and other forms of TEAS compared with the treatment group, it will be excluded.

2.2.4. Types of outcome measures. The primary outcomes consists of the indicator of the pain intensity, such as the visual analog Ssale (VAS) and the consumption of postoperative analgesics. As we all known, pain intensity is considered an indispensable statistical indicator in pain assessment and management,[8] and VAS is commonly used in this area. The secondary measures are the postoperative quality of life, the duration of hospitalization, and the incidence of adverse reactions and serious events. For the postoperative quality of life, the quality of recovery-40 has been proved to be an important indicator to evaluate postoperative recovery in clinical practice.[9].

2.3. Data sources

2.3.1. Electronic searches. Our study will perform a comprehensive and systematic literature search mainly on 7 electronic databases from their inception up to November 30, 2020. The specific databases are as following: PubMed, the Cochrane Library, Embase, China National Knowledge Infrastructure, Chongqing VIP Information, and WanFang Data, and Chinese Biomedical Database. Medical Subject Heading terms and free-text terms with logical operators will be adopted in the strategies of researches. Asterisks, regarded as truncation symbols, will play an important role in searching for all designs with asterisks. The retrieval strategy in PubMed is considered as an example in the researching process, which is presented in Table 1.

2.3.2. Searching for other resources. The ongoing or unpublished studies relevant to our study will be searched from PROSPERO, ClinicalTrials.gov, Chinese Clinical Trial Registry, the US National Institutes of Health register, the World Health Organization International Clinical Trials Registry Platform. We will also conduct manual retrieval of relevant trials in books, journals, academic reference reporting, and the field of potential gray literature.

2.4. Data collection and analysis

2.4.1. Selection of studies. All the literature retrieved from the databases will be imported into the Document Explorer (EndNote X9 software) and then complete the selection process of repeated literature automatically. Two researchers (DM and MY) will read the titles and abstracts of the literature to eliminate the irrelevant and then communicated with each other. Further full-texts will be downloaded and selected whether it is consistent with our included criteria. The disagreements in any of the above processes, the 2 researchers (DM and MY) will communicate with the third party (SQ) for a final judgment. The flow chart of the selection procedure followed by Preferred Reporting Items for Systematic Reviews and Meta-Analysis Protocol (PRISMA-P) was shown in Figure 1.

2.4.2. Data extraction. Two independent investigators will be responsible for extracting and tabulating the data regarding the

Table 1

Search strategy of PubMed.

Number	Search items
1	transcutaneous electrical acupoint stimulation [ti, ab]
2	transcutaneous acupoint electrical stimulation [ti, ab]
3	electr* stimulat* [ti, ab]
4	electr* acustimul* [ti, ab]
5	electroacupuncture* [ti, ab]
6	electro-acupuncture [ti, ab]
7	TEAS [ti, ab]
8	1 or 2 or 3 or 4 or 5 or 6 or 7
9	Laparoscopy [mh]
10	laparoscop* [ti, ab]
11	coelioscop* [ti, ab]
12	celioscop* [ti, ab]
13	peritoneoscop* [ti, ab]
14	9 or 10 or 11 or 12 or 13
15	Pain, Postoperative [mh]
16	postoperative pain [ti, ab]
17	postoperative analgesi* [ti, ab]
18	pain management [ti, ab]
19	ache* [ti, ab]
20	suffering* [ti, ab]
21	Discomfort [ti, ab]
22	15 or 16 or 17 or 18 or 19 or 20 or 21
23	8 AND14 AND 22

scheduled program. Discrepancies will be resolved via communicating and deciding with the third party (YC). The necessary e-mails or telephones with the correspondent authors will be conducted when the data are incomplete or ambiguous. In the multi-arm RCTs, our study will only extract the data related to our criteria according to the specific interventions.

The predesigned data extraction items: first author name, publication year, types of surgery, sample sizes, interventions, intervention measures and time, acupoints, waveform, electrical stimulation frequency, outcome measures, and adverse events.

2.4.3. Dealing with missing data. We will resort to the corresponding author of the literature via e-mail or telephone for the missing or inexplicit data. If no response is received from the author or the data is confirmed to have been lost, we will exclude the relevant literature. The process of finding data will be described in detail in the discussion section.

2.4.4. Assessment of risk of bias in included studies. Our study will assess of risk of bias in included studies following the Cochrane risk of bias tool,[10] which is consisted of random sequence generation, allocation concealment, blinding of participants and personnel, blinding of outcome assessment, selective reporting, incomplete outcome data, and other risks of bias, in which the "other risks of bias" are included as following:

(1) Whether the reported baseline between the treatment group and the control group is comparable.
(2) Whether the criteria for inclusion or exclusion are clear.
(3) Whether the experimental design is clinical or feasible.
(4) Whether there is a crucial conflict of interest that leads to increased bias.

The level of the assessment can be divided to 3 parts called "low-risk," "high-risk," or "unclear-risk." Disagreements will be resolved via communications between the 2 investigators or after discussion with a third party (YM and ZQ).

2.4.5. Statistical analysis. Statistical analysis of the included RCTs will be performed with the Stata 15.0 software. The mean difference (MD) or standard MD with 95% confidence interval (CI) was used for continuous variables. The relative risk with 95% CIs was utilized for dichotomous variables. In terms of the multi-arm RCTs that met the included criteria, meta-analysis will merge the data into a unified intervention group prudently. The magnitude of heterogeneity will be measured using the heterogeneity I^2 statistic indicator. Different data processing models will be used according to different data results: a fixed-effects model will be used for pooled data when $I^2 < 50\%$ and a random-effects model will be used when $I^2 \geq 50\%$. The chi-squared statistic will be performed to detect a heterogeneity test for each merged analysis. If $I^2 \geq 50\%$, the synthesized studies will be considered as an indicator of high heterogeneity. When significant heterogeneity exists in the results of the combined data, sensitivity analysis will be used to assess the stability of the results of each meta-analysis and help identify the sources of heterogeneity by screening the literature with a higher risk of bias. Descriptive analysis will be conducted when meta-analysis is not appropriate.

2.4.6. Subgroup analysis. Subgroup analysis will be performed to identify the possible factors that contributed to the heterogeneity. The influencing factors can be divided into the following categories:

(1) Different intervention duration.
(2) Different interventions.
(3) Different types of surgery.
(4) Different waveforms or frequencies of electrical stimulation.

2.4.7. Assessment of reporting biases. Funnel plots will be performed to evaluate the publication bias if there are enough amounts of RCTs ($n \geq 10$) in each of the meta-analyses. An Egger regression test will be used to quantitatively evaluate funnel plot asymmetry.[11]

2.5. Paper writing

The protocol of our study will follow the PRISMA-P) statement guideline.[12] Besides, our study will be performed in compliance with the PRISMA statement guidelines.[13]

2.6. Quality of the evidence

The Grading of Recommendations Assessment, Development, and Evaluation approach will be conducted to evaluate the quality of evidence for outcomes.[14] And the level of evidence will be divided into 4 categories: very low, low, moderate, and high.

3. Discussion

As far as we know, shoulder and back pain, subdiaphragmatic pain, intercostal muscle pain, and other non-surgical incision pain associated with laparoscopic surgery are more serious postoperative complications of this surgery, which is called the "Laparoscopic Postoperative Pain Syndrome."[15,16] Although there are many methods of postoperative pain management, there

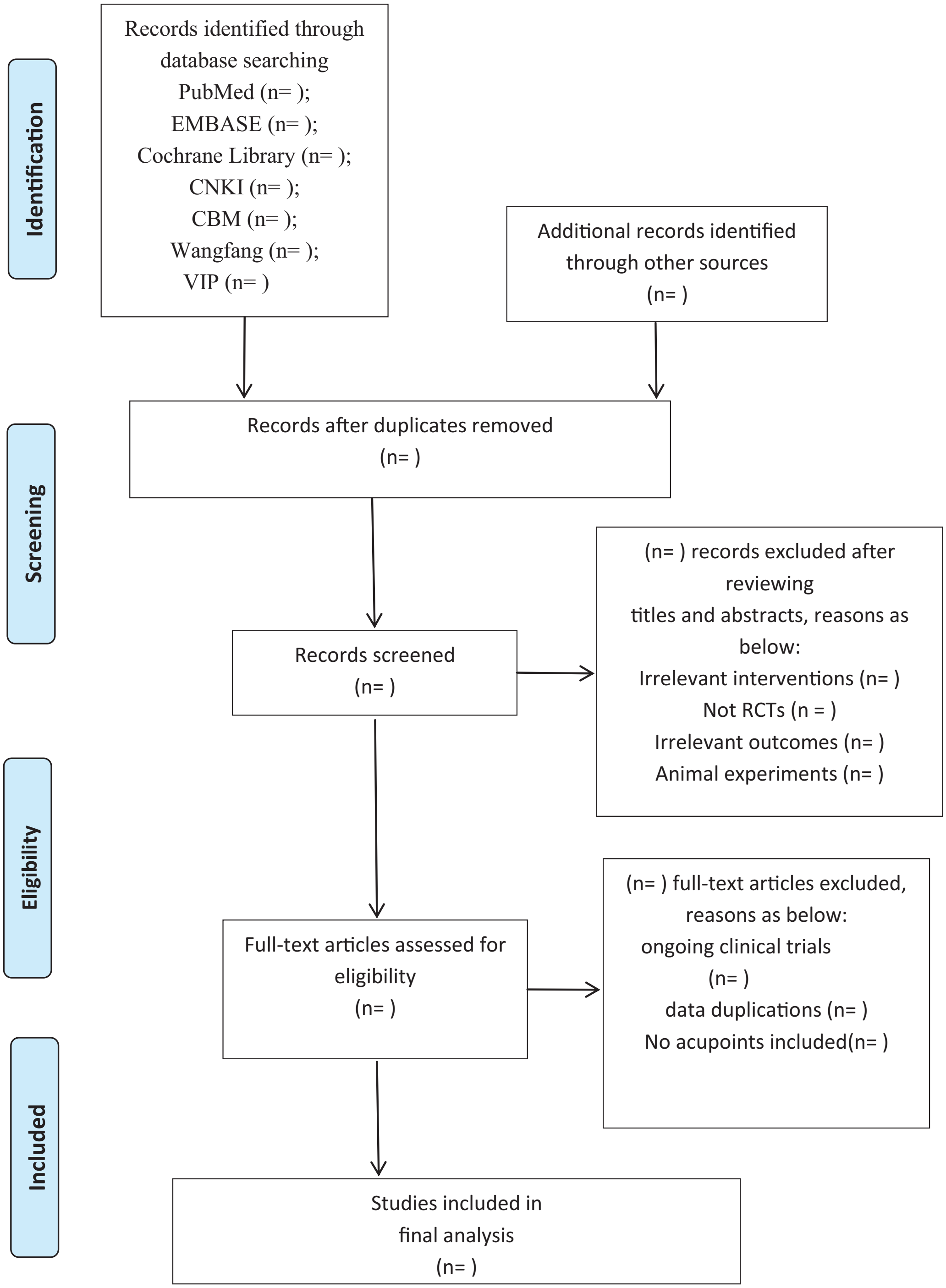

Figure 1. PRISMA flow diagram of the study process. PRISMA=Preferred Reporting Items for Systematic Reviews and Meta-Analysis.

are still some side effects and adverse reactions. Therefore, to reduce the side effects of drugs, enhance patients' satisfaction with treatment and improve postoperative recovery, the advantages of TEAS in postoperative pain management have been gradually manifested. Both patients and caregivers could participate in the clinical application of TEAS after the short-time but accurate training. Besides, the equipment can be reused, and patients' treatment compliance is relatively high. More and more clinical RCTs report the efficacy of TEAS in postoperative laparoscopic pain management. Our study is the first meta-analysis of the efficacy and safety of TEAS for postoperative laparoscopic pain management, aiming to provide strong evidence for clinical support.

Author contributions

Conceptualization: Dan Meng, Yifei Mao, Yongmei Song.
Data curation: Dan Meng, Yifei Mao, Quanmei Song.
Investigation: Dan Meng, ChunChun Yan, Qinyu Zhao.
Methodology: Dan Meng, Yifei Mao, Mengqi Yang.

Supervision: Dan Meng, Yongmei Song.
Validation: Dan Meng, Yifei Mao, Yongmei Song.
Visualization: Dan Meng, Yongmei Song.
Writing – original draft: Dan Meng, Yongmei Song.
Writing – review & editing: Dan Meng, Yongmei Song.

References

[1] Kehlet H, Slim K. The future of fast-track surgery. Br J Surg 2012; 99:1025–6.
[2] Cannesson M, Kain Z. Enhanced recovery after surgery versus perioperative surgical home: is it all in the name. Anesth Analg 2014;118:901–2.
[3] Bruera E, Paice JA. Cancer pain management: safe and effective use of opioids. Am Soc Clin Oncol Educ Book 2015;e593–9.
[4] Wu MS, Chen KH, Chen IF, et al. The efficacy of acupuncture in post-operative pain management: a systematic review and meta-analysis. PLoS One 2016;11:e0150367.
[5] Mi ZH, Gao J, Chen XP, Ge Y, Lu K. Effect of transcutaneous acupoint electrical stimulation on the quality of early postoperative recovery in patients undergoing laparoscopic cholecystectomy. Chin Acupuncture Moxibustion 2018;38:256–60.
[6] Huang W, Long W, Xiao J, Zhao G, Yu T. Effect of electrically stimulating acupoint, Zusanli (ST 36), on patient's recovery after laparoscopic colorectal cancer resection: a randomized controlled trial. J Tradit Chin Med 2019;39:433–9.
[7] Guo YQ, Wu W, Yang ZL, et al. Effect of transcutaneous acupoint electrical stimulation combined with general anesthesia on complications after laparoscopic ovarian cyst resection. J Liaoning Univ Tradit Chin Med 2016;18:100–2.
[8] Fainsinger RL, Fairchild A, Nekolaichuk C, Lawlor P, Lowe S, Hanson J. Is pain intensity a predictor of the complexity of cancer pain management? J Clin Oncol 2008;27:585–90.
[9] Gornall BF, Myles PS, Smith CL, et al. Measurement of quality of recovery using the QoR-40: a quantitative systematic review. Br J Anaesth 2013;111:161–9.
[10] Higgins JPT, Thomas J, Chandler, et al. Cochrane Handbook for Systematic Reviews of Interventions version 6.0 (updated July 2019). Cochrane, 2019. Available from www.training.cochrane.org/handbook.
[11] Egger M, Davey Smith G, Schneider M, Minder C. Bias in meta-analysis detected by a simple, graphical test. BMJ 1997;315:629–34.
[12] Shamseer L, Moher D, Clarke M, et al. Preferred reporting items for systematic review and meta-analysis protocols (PRISMA-P) 2015: elaboration and explanation. BMJ 2015;349(jan02 1):g7647.
[13] Moher D, Liberati A, Tetzlaf J, Altman D. Preferred reporting items for systematic reviews and meta-analyses: the PRISMA statement. Int J Surg 2010;8:336–41.
[14] Balshem H, Helfand M, Schünemann HJ, et al. GRADE guidelines: 3. Rating the quality of evidence. J Clin Epidemiol 2011;64:401–6.
[15] Chen LZ. The shadow response of functional rehabilitation exercise to the syndromes of pain after abdominal endoscopic surgery in gynecology. Medical Forum 2010;17:165–9.
[16] Yao QE. Nursing progress of non-incision pain after gynecological laparoscopy. Contemporary Nurse 2012;2:7–9.
